# Strychnine in Typhoid Fever

**Published:** 1861-02

**Authors:** 


### Strychnine in Typhoid Fever.

Prof. N. S. Davis of Chicago, in a clinical lecture reported for the *Chicago Medical Examiner*, advocates the use of strychnine in typhoid fever. In cases having a tendency to prostration rapidly, the Doctor found the remedy particularly useful. A case is mentioned in which alcohol, turpentine, quinine &c., had been used without arresting the disposition to prostration, when strychine was administered with the most satisfactory result.

The following is the combination in which it was given:

℞. Strychine, gri.

Nitric acid, ʒ i.

Tinct. opii, ʒ ii.

Water, ℥ ii.

A teaspoonful to be given every four hours.

